# A self-reference false memory effect in the DRM paradigm: Evidence from Eastern and Western samples

**DOI:** 10.3758/s13421-018-0851-3

**Published:** 2018-08-23

**Authors:** Jianqin Wang, Henry Otgaar, Mark L. Howe, Chu Zhou

**Affiliations:** 1 Department of Clinical Psychological Science, Faculty of Psychology and Neuroscience, Maastricht University, PO Box 616, 6200 MD Maastricht, The Netherlands; 2 City, University of London, London, UK; 3 Fudan University, Shanghai, China

**Keywords:** Self-reference, False memory, Net accuracy, Spreading activation, Fuzzy-trace theory

## Abstract

It is well established that processing information in relation to oneself (i.e., self-referencing) leads to better memory for that information than processing that same information in relation to others (i.e., other-referencing). However, it is unknown whether self-referencing also leads to more false memories than other-referencing does. In the current two experiments with European and East Asian samples, we presented participants the Deese–Roediger–McDermott lists together with their own name or other people’s name (i.e., “Trump” in Experiment 1 and “Li Ming” in Experiment 2). We found consistent results across the two experiments; that is, in the self-reference condition, participants had higher true and false memory rates compared with those in the other-reference condition. Moreover, we found that self-referencing did not exhibit superior mnemonic advantage in terms of net accuracy compared with other-referencing and neutral conditions. These findings are discussed in terms of theoretical frameworks such as spreading activation theories and the fuzzy-trace theory. We propose that our results reflect the adaptive nature of memory in the sense that cognitive processes that increase mnemonic efficiency may also increase susceptibility to associative false memories.

**Electronic supplementary material:**

The online version of this article (10.3758/s13421-018-0851-3) contains supplementary material, which is available to authorized users.

The self plays an important role in memory (Conway, 2005; Howe, 2014; Klein, 2012). It is well established that information is better remembered when it is processed in relation to the self than when it is processed in relation to other sources (Symons & Johnson, 1997). For instance, judging words for their personal relevance (“Does this word describe you?”) produces superior memory recall than does other-referencing (“Does this word describe Henry?”) or semantic encoding (“Does this word mean the same as XXX?; Kuiper & Rogers, 1979; Rogers, Kuiper, & Kirker, 1977). The superior mnemonic effect for items encoded with respect to the self has been termed as the self-reference effect (SRE) and has received considerable empirical support. For instance, SRE has been found in various age groups (from children to old people; Cunningham, Brebner, Quinn, & Turk, 2014; Gutchess, Kensinger, Yoon, & Schacter, 2007) as well as in different cultures (Zhu, Zhang, Fan, & Han, 2007).

One of the proposed mechanisms underlying SRE is relational processing or organizational processing (Klein, 2012; Klein & Loftus 1988; Symons & Johnson, 1997). Relational processing refers to the encoding of associations among items in a list leading participants to think about the shared concept or label (Klein, 2012; Klein & Loftus, 1988). For example, when participants see the words *sound* and *piano*, the items can be organized as concepts related to “music.” Another example would be items sharing the same category, such as *dog* and *horse* that can be both processed under the category “animal.” Klein & Loftus, (1998) manipulated the relatedness among items within a list and found that for weakly related lists, category sorting (i.e., relational processing) produced equivalent recall as self-referenced processing, suggesting relational processing is one of the mechanisms underlying SRE.

In line with this, Sui and Humphreys (2015) have recently proposed that self-reference increases the binding among different forms of information in memory. For instance, evidence shows that people not only remember the self-referenced stimuli better than other-referenced stimuli but also remember the stimuli–self associations (e.g., an apple paired with one’s own face) better than the stimuli–other associations (e.g., a cup paired with other people’s face; Cunningham et al., 2014). Leshikar and Duarte (2014) reported that both young and old adults exhibited better relational memories for object–scene bindings (e.g., saxophone–beach) in the self-reference rather than the other-reference condition. According to Sui and Humphreys, the self acts like a form of “associative glue” in a memory network, and when self-representations are activated, they strengthen the binding among different stimuli in relation to the self-representation, thus increasing retention for experienced stimuli. Taken together, research so far has demonstrated that relational processing among the list items or the binding among stimuli may account for the mnemonic superiority of self-referenced processing.

Interestingly, according to spreading activation theories of false memory (e.g., activation association theory [AAT]: Howe, Wimmer, Gagnon, & Plumpton, 2009; activation/monitoring theory [AMT]: Roediger, Balota, & Watson, 2001a), relational processing or binding between items may also contribute to the creation of false memories. False memories refer to memory for details or events that were not experienced. The Deese–Roediger–McDermott paradigm (DRM; Deese, 1959; Roediger & McDermott, 1995) is a typical experimental paradigm used to study false memories. Here, participants study lists of associated words (e.g., *sound, piano, sing, radio, band, melody, horn, concert, instrument*), and during memory tests, participants oftentimes falsely recall or recognize the presence of a nonpresented critical lure (i.e., *music*) that is associated with the list items. Studies with the DRM paradigm show that increasing the associative strength of a studied list (i.e., increasing relational processing) results in higher levels of false memories (Gallo & Roediger, 2002; Howe et al., 2009; for results showing that the associative strength of a studied list is not the source of false-memory effects, see Cann, McRae, & Katz, 2011). Following this line of reasoning, enhancing relational processing using self-referencing should not only make people better remember what has been presented but should also enhance the susceptibility to the DRM memory illusion compared with other-referencing conditions. The current experiments were conducted to examine this idea.

Spreading activation theories (e.g., Howe et al., 2009) suggest that related concepts, such as the DRM list items, are embedded within an associative network and that the presence of an item (e.g., the word *piano*) activates a corresponding concept (see also Otgaar, Muris, Howe, & Merckelbach, 2017). Most importantly, according to these theories, activation will automatically spread to “nearby” related concepts (e.g., *music*) and the level of activation determines the rate of recall/recognition (Anderson, 1983). The activation of related but nonpresented concepts leads to false recollections of nonpresented critical lures. As Fig. 1 shows, based on mechanisms of false memory and SRE, when the DRM list items are processed in relation to the self, the self may strengthen the relatedness among the items as well as the activation spreading to the critical lures, thus evoking more false memories of the critical lures than when processing information related to others.

**Fig. 1:**
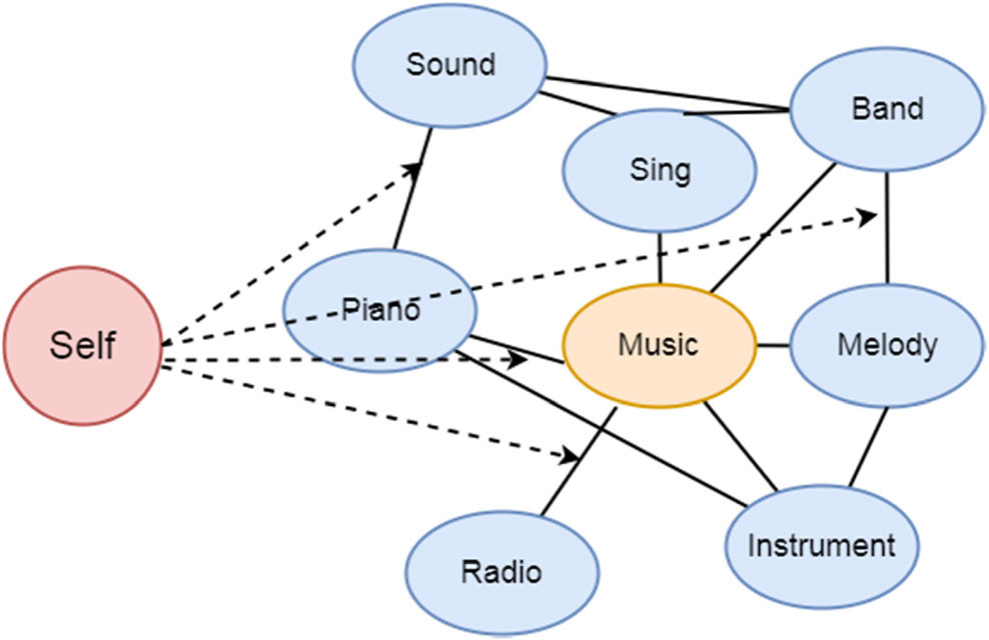
Hypothetical associative memory network when the self is referenced, based on spreading activation theories (Howe et al., 2009; Roediger et al., 2001a) and the mechanisms of SRE (Klein, 2012; Sui & Humphreys, 2015). Blue circles represent DRM list items; yellow circle represents the critical lure; solid lines represent relations among items, and dashed arrows represent the strengthening effect to the relatedness among items. (Color figure online)

In addition to spreading activation theories, another theoretical account, fuzzy-trace theory (FTT; Brainerd & Reyna, 2002; Brainerd, Reyna, & Ceci, 2008a), also predicts that the self may increase false memory. According to FTT, when encoding an item, verbatim traces and gist traces of the item are stored in memory. Verbatim traces are item-specific features (e.g., unique spelling of a studied word) that distinguish one item from another. Gist traces are memory representations of semantic and relational information (Brainerd & Reyna, 2005). Brainerd, Yang, Reyna, Howe, and Mills (2008b) showed that relations between studied items and critical lures are mostly semantic in the DRM paradigm. When a DRM list including *dog, cat, horse*, and *pig* is presented, participants may extract a gist such as *animal* that semantically relates to all items (i.e., all items belong to *animal*). Hence, participants may confuse *animal* with presented items and falsely recognize it as presented since it shares gist (i.e., meanings, with presented items). Thus, gist processing supports false memory according to FTT (Brainerd & Reyna, 2002).

Of relevance here is that when self-reference facilitates the *semantic* relatedness among DRM items, it actually facilitates gist extraction according to FTT. Because gist traces support false memory of critical lures, self-referencing may increase false memory rates compared with other-referencing. FTT elaborates the mechanism of how relational processing impacts false memory from a different perspective than associative theories. That is, FTT proposes that relational processing benefits false memory via encouraging gist processing, whereas associative theories propose that relational processing facilitates false memory via strengthening associations and activations. Nonetheless, both theoretical approaches predict that self-reference would increase false memory through increasing relational processing.

Indeed, there is some evidence suggesting that self-reference may be related to false memories. Rogers, Rogers, and Kuiper (1979) found that false alarms for personal adjectives increased as the degree of self-reference increased. Rosa and Gutchess (2013) replicated this effect and extended this effect to old adults (see also Rosa, Deason, Budson, & Gutchess, 2015). They asked participants to rate adjectives (e.g., cultured, sensible) for self-descriptiveness on a 9-point scale and later measured their memories for the adjectives and some adjective lures. The authors found that high self-descriptiveness words led to higher false alarms than low self-descriptiveness words did. One limitation with the aforementioned research is that they did not compare the false alarms between the self-reference and other-reference conditions, but only examined the relationship between the degree of self-reference and false alarms. Furthermore, previous studies did not use a well-established false memory paradigm (e.g., the DRM paradigm) known to elicit false memories that might be caused by similar mechanisms as the SRE. Choosing such a paradigm would enable us to test the mechanisms underlying SRE and false memories.

To date, no study has compared the false memories of self-reference versus other-reference conditions, and no study has examined the relationship between true and false memories (i.e., net accuracy) generated from self-referential processing. Net accuracy measures the overall memory accuracy, which is the ratio of true memories to true memories plus false memories (Brainerd et al., 2008a). Because relational processing may amplify both true memories and false memories, it is unknown whether self-referencing would increase or even decrease the net accuracy (i.e., true memory to true memory plus false memory) relative to other-referencing. However, since self-referencing has been demonstrated to be a superior memory encoding strategy in previous literature, one would expect that self-referencing should increase the overall memory accuracy or net accuracy. The present study aimed to examine self-referenced false and true memories as well as net accuracy in the DRM paradigm.

## The current experiments

We presented participants with DRM lists appearing together with either their own name (self-referenced condition) or some other people’s name (i.e., Trump; other-referenced condition). During the study phase, participants were asked to remember the words shown to them as well as with which source (self or other) the words appeared together. After a filler task, they were asked to recognize the words as presented or not in the recognition phase. Based on previous research (e.g., Rogers et al., 1979; Rosa & Gutchess, 2013) as well as relational processing mechanism of SRE, we predicted that the self-referenced condition would result in higher false recognition rates of the critical lures than the other-referenced condition. We also predicted that self-referencing would lead to the highest true recognition rate of the studied items (i.e., the SRE).

Importantly, previous research has shown that self-reference can increase memory for perceptual details of the studied items (Hamami, Serbun, & Gutchess, 2011; Leshikar, Dulas, & Duarte, 2015; Serbun, Shih, & Gutchess, 2011). Serbun et al. (2011) asked participants to encode objects in relation to self or other and found that self-referenced objects were remembered with more visual details. Leshikar et al. (2015) used the remember/know (R/K) procedure to measure recollection and familiarity of self-referential memories. Recollection and familiarity are two subtypes of recognition memory which can be calculated from R/K responses. Recollection indicates remembering episodic details, while familiarity refers to the feeling of recognizing an item but the specific details (Yonelinas, 2002). Leshikar et al. (2015) found that the self-referencing condition exhibited better recollection of studied items than semantic encoding (i.e., processing the meaning of presented words). They also found more phenomenological details on the Memory Characteristic Questionnaire (MCQ) measurements. It is unknown whether self-referencing would also increase the phenomenology of false memories. It might be that self-referencing impacts familiarity of falsely recalled items, as Rosa and Gutchess (2013) suggested. However, it is also possible that self-referencing impacts true and false memories in a similar way by increasing recollection, as they share the same activation network. We used the R/K judgment procedure (Yonelinas, 2002; Yonelinas & Jacoby, 1995) in the recognition phase to examine the recollection and familiarity of critical lures in self-reference versus other-reference conditions.

## Experiment 1

### Method

#### Participants

With an estimation of medium effect size (*d* = 0.5; Symons & Johnson, 1997) and power of 0.8, a priori power analysis using G*Power 3.1 (Faul, Erdfelder, Lang, & Buchner, 2007) showed that 34 participants were needed. Thirty-nine participants from Maastricht University, The Netherlands, were tested. The sample consisted of eight males and 31 females (*M*_age_ = 21.6, *SD* = 2.29, range: 18–31 years). All participants were fluent in English. The experiment was approved by the ethical board of Faculty of Psychology and Neuroscience, Maastricht University.

#### Materials

Eighteen DRM word lists with 12 words per list were used. The lists have been used in previous work as well (e.g., Howe, Garner, & Patel, 2013). DRM lists were pseudorandomly assigned to the self-reference (six lists, mean BAS = 0.26, *SD* = .13), other-reference (six lists, Mean BAS = 0.26, *SD* = .12), and control conditions (six lists, mean BAS = 0.26, *SD* = .10), with the backward association strength (BAS) matched across three conditions, *F*(2, 16) = 0.006, *p* = .995. Besides controlling the BAS across conditions, we switched the lists in the self-reference and other-reference conditions for half of the participants (*n* = 19) to eliminate any list effects. The recognition list included 18 critical lures (one critical lure per list), 36 studied items, and 36 unrelated new items. The studied items were from the third and 10th position of each list.

#### Design and procedure

The experiment used a 3 (reference: self vs. other vs. neutral) × 2 (memory type: true vs. false) within-subjects design. All participants were tested individually in a quiet and isolated room. The experiment was programmed using Visual Basic.

**Fig. 2:**
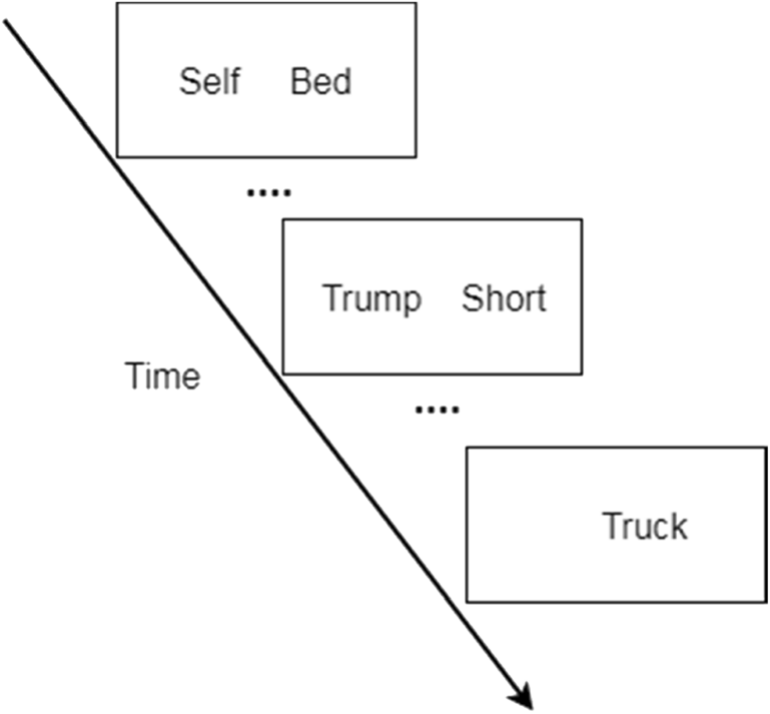
Illustration of one block in the study phase (“Self” stands for participant’s own name). *Bed*, *Short* and *Truck* are representative words from different lists. Each word pair was presented for 1,500 ms with 500-ms interstimulus interval

In the study phase, participants first were asked to fill in some information including their own name (the name that they were most frequently referred to). Then the DRM words appeared one by one together with either their own name (self-reference condition) or the name “Trump” (other-reference condition), or the DRM words appeared alone (neutral condition). “Trump” was used in the other-reference condition as it was a very familiar name due to the American election at the time of our data collection. Participants were asked to remember the words and to which source they appeared together with (self, Trump, or alone). The DRM words were shown list by list, and the items within a list appeared with the same source (see Fig. 2). All lists were presented in English as our previous work showed that samples from Maastricht University did not differ from native English speakers in terms of DRM false recognition rates (Wang, Otgaar, Howe, Smeets, & Merckelbach, 2017). The experiment was separated in six blocks with each block containing three BAS matched lists (a self-referenced list, an other-referenced list, and a control list). The sequence of the lists within a block was randomized.

After the study phase, participants performed a filler task (playing the “Bejeweled” game) for around 5 min. Then, their memories were tested using a recognition task. Words were shown at the center of the screen, and participants responded by clicking the “Remember,” “Know,” or “New” button. Participants received the following R/K instructions adapted from previous research (see Yonelinas, 2002; Yonelinas & Jacoby, 1995):

“*In the next task, words will be shown at the center of the screen. Some words were presented in the previous phase and some were not. Your task is to decide whether the words are old (i.e., presented words) or new (i.e., not presented).*

*If it is a new word, click the “New” button. If it is an old word: When you clearly remember that the item was presented (i.e., you can recall specific information such as size, font, etc.), click the “Remember” button; when you know the item was presented but you cannot recall specific details, click the “Know” button.*” Three example words were presented first to help participants understand the instructions.

### Results and discussion

#### Recognition rates

“Remember” and “Know” responses were combined together when calculating the overall “old” recognition response rate. False memory was defined as falsely recognizing a critical lure as old. The mean false alarm rate of unrelated items was 19.66% (95% CI [14%, 25%]), and it was statistically lower than false recognition rates of critical lures in any condition (*p*s < .001), showing a typical false memory effect.

**Fig. 3:**
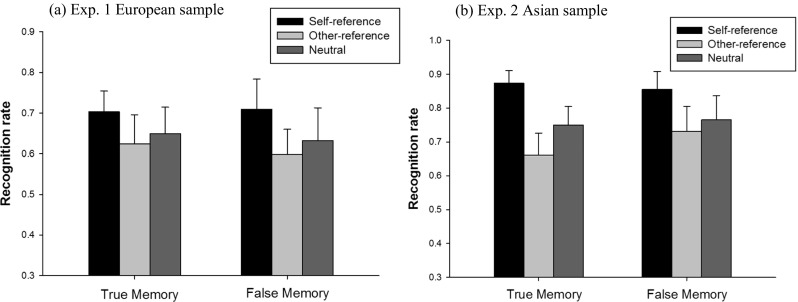
True and false recognition rates in self-reference, other-reference, and neutral conditions in (**a**) Experiment 1 (European sample; *n* = 39) and (**b**) Experiment 2 (East Asian sample; *n* = 29) (Error bars stand for 95% CIs)

A 3 (reference: self vs. other vs. neutral) × 2 (memory type: true vs. false) repeated-measures ANOVA was conducted on recognition rates. We found a statistically significant main effect of reference, *F*(2, 76) = 6.77, *p* = .002, partial η^2^ = 0.15. Pair-wise comparisons with Bonferroni corrections indicated that the self-reference condition had higher true and false recognition rates than the other-reference condition did (*p* < .001), and the neutral condition did not differ from the other-reference condition (*p* > .80). There was no statistically significant main effect of memory type, *F*(2, 76) = 0.22, *p* = .65, indicating equivalent level of true memories and false memories were recognized. No statistically significant interaction effect between reference and memory type was found, *F*(2, 76) = 0.21, *p* = .81, suggesting that the SRE is similar for both true and false memories (see Figure 3a). The raw recognition rates in each condition were presented in the Supplementary Materials.

We were specifically interested in comparing the effect sizes of SRE in false memories and true memories, respectively. The result showed that false recognition rates in the self-reference condition were significantly higher than in the other-reference condition, *t*(38) = 3.68, *p* = .001, Cohen’s *d* = 0.60. The self-reference condition also had significantly higher false recognition rates than the neutral condition using a one-tailed t test, *t*(38) = 1.92, *p* = .03, Cohen’s *d* = 0.31. A paired-samples *t* test showed significantly higher true memory rates in the self-reference condition than in the other-reference condition as well, *t*(38) = 2.02, *p* = .05, Cohen’s *d* = 0.33.

#### Net accuracy

Net accuracy was calculated as the ratio of true recognition to true recognition plus false recognition (Brainerd et al., 2008a). We conducted a repeated-measures ANOVA to compare net accuracy in the self-reference, other-reference, and neutral conditions. No significant main effect was found on the reference variable, *F*(2, 76) = 0.10, *p* = .91. Net accuracy of the self-reference processing (*M* = 0.51, 95% CI [0.47, 0.54]) did not differ from that of the other-reference processing (*M* = 0.50, 95% CI [0.46, 0.55]) or the neutral condition (*M* = 0.51, 95% CI [0.48, 0.55]). The results suggest that self-referencing did not increase the overall accuracy of memory compared with other-referencing and the neutral conditions in the DRM paradigm.

#### Recollection and familiarity of SR false memory

Recollection is the rate of “remember” responses, whereas familiarity is calculated as the rate of”knowing” responses divided by 1 minus the rate of remember responses (Knowing rate/(1 − recollection rate); Yonelinas, 2002; Yonelinas & Jacoby, 1995). We conducted repeated-measures ANOVAs to compare recollection of critical lures in different reference conditions. As Table 1 shows, we found that different reference conditions led to statistically different recollection rates for critical lures, *F*(2, 76) = 7.24, *p* = .001, partial η^2^ = 0.16. Follow-up Bonferroni contrasts showed that in the self-reference condition, critical lures received statistically higher recollection scores than in the other-reference condition, *t*(38) = 3.55, *p* = .001, Cohen’s *d* = 0.58, and neutral conditions, *t*(38) = 2.97, *p* = .005, Cohen’s *d* =0.48. For familiarity, all conditions had equal levels of familiarity, *F*(2, 76) = 1.75, *p* = .18. Thus, processing information related to oneself mainly increased false recollections relative to the condition in which information was related to others.

**Table 1:** Recollection and familiarity of critical lures and studied items in different reference conditions (means with 95% CIs)

Reference	Critical lures		Studied items	
	Recollection	Familiarity	Recollection	Familiarity
Self-reference	0.49 [0.40, 0.57]	0.44 [0.34, 0.55]	0.48 [0.41, 0.56]	0.42 [0.34, 0.49]
Other-reference	0.35 [0.28, 0.43]	0.36 [0.27, 0.44]	0.39 [0.32, 0.47]	0.40 [0.32, 047]
Neutral	0.35 [0.27, 0.44]	0.45 [0.35, 0.56]	0.37 [0.30, 0.44]	0.45 [0.37, 0.53]

#### Recollection and familiarity of SR true memory

We conducted the same repeated-measures ANOVAs on recollection and familiarity of true memories. We found a statistical difference in recollection scores for presented items among the reference conditions, *F*(2, 76) = 4.97, *p* = .009, partial η^2^ = 0.12, while familiarity was not significantly impacted by reference, *F*(2, 76) = 0.65, *p* = .52. Paired-samples *t* tests showed that in the self-reference condition, presented items had statistically higher recollection scores than in the other-referenced condition, *t*(38) = 2.07, *p* = .045, Cohen’s *d* = 0.33, and the neutral condition, *t*(38) = 3.20, *p* = .003, Cohen’s *d* = 0.51. The latter two did not differ from each other, *t*(38) = 0.75, *p* = .46. The results were consistent with previous studies which found that self-reference increased recollections of true memories (Leshikar et al., 2015).

In Experiment 1, we showed that in line with our hypothesis, relating information to oneself increased both true and false memories compared with relating information to others. Moreover, we found that self-reference specifically increased recollection scores of studied items and critical lures. These may be explained by our hypothetical mechanism of the SRE in the associative memory network (see Fig. 1). Because the self facilitates the relational processing or the binding of DRM list items (Klein, 2012; Sui & Humphreys, 2015), on the one hand, facilitated relatedness increases the phenomenological level of activated recollection of the studied items, as demonstrated by our results and also previous SRE research (e.g., Leshikar et al., 2015; Serbun et al., 2011). On the other hand, the increased activation may automatically spread to critical lures (e.g., AAT; Howe et al., 2009), and increase the activation level of critical lures as well. As a result, the highest proportion of critical lures that were falsely remembered was in the self-reference condition.

Besides relational processing, the other mechanism that may mediate SRE is item-specific processing (or elaboration; Klein, 2012). Item-specific processing refers to encoding specific information of an item (Hunt & Einstein, 1981; Klein & Loftus, 1988). For example, for *sound* and *piano*, *sound* can be encoded as something people hear, while *piano* is an object. However, item-specific processing distinguishes different items, and it has been found to reduce false memories (e.g., Arndt & Reder, 2003; Israel & Schacter, 1997; McCabe, Presmanes, Robertson, & Smith, 2004). For instance, when DRM items within a list were presented in different fonts, false memories were reduced compared with when DRM items were presented in the same font (Arndt & Reder, 2003). Our results showing that self-referencing increased false memories seemed not to support item-specific processing as the dominant processing in the current paradigm. Moreover, if item-specific processing is the main mechanism of SRE here, one would expect it to increase net accuracy as it should increase true memory but decrease false memory, which was not the case in our results.

Surprisingly, we found that net accuracy of self-referencing did not differ significantly from other-referencing and neutral conditions, suggesting self-referencing does not increase the overall memory accuracy in a paradigm where relational processing would lead to associative memory illusions. The results showed that self-referencing increased true and false memories to the same extent compared with the other conditions, leading to no statistical difference between conditions. These results indicate that relational processing is the dominant processing in SRE with the DRM paradigm. Relational processing results in an increase of activation of both studied items and critical lures that share the same memory network.

Thus, Experiment 1 shows that processing information in relation to the self elevates true *and* false memory levels. One limitation with the current sample is that most participants were female. One may also wonder that the difference between the self-reference condition and the neutral condition on false recognition rates reached only one-tail significance. However, we had predicted a one directional effect; that is, self-reference increases false memory relative to the other conditions, and our prediction was supported by the results. Moreover, this will not hinder our conclusion on SRE as SRE here is based on self-referencing versus other-referencing comparisons. Another interesting observation was that net accuracy was not affected by self-referencing. To examine the reliability of this finding, we reanalyzed data from another sample in Experiment 2, to test whether similar findings would be found even under a different cultural background. Specifically, in Experiment 2, a similar approach was used in an Asian sample, and there was a neutral reference (a color square) in the neutral condition.

## Experiment 2

Self-representation is impacted by cultural context. For instance, Westerners normally value the self as independent and less connected to others while East Asians are more likely to value the relationship between the self and others (Markus & Kitayama, 1991). Zhu et al. (2007) found that processing information in relation to one’s mother activated similar neural correlates as self-referenced processing in Asian participants but not in Western subjects, suggesting culture shapes neural correlates of self-representational cognitive processes. Other research found that Eastern Asians tend to focus more on the relationships and contexts when processing pictures such as a lion standing in a desert background, while Westerners focus more on the objects such as the lion (Goh et al., 2007; Nisbett & Masuda, 2003). Data from East Asian participants were analyzed in Experiment 2 to explore whether there was any cultural difference in self-referenced true and false memories as well as net accuracy in the DRM paradigm. Since East Asians pay more attention to relationships with others, processing information in relation to others may already show a high level of relational processing. We predicted that East Asians might not exhibit a self-reference false memory effect as strong as Europeans, and thus net accuracy (i.e., true memory to true memory plus false memory) should be larger in East Asians.

### Method

#### Participants

Twenty-nine participants from Fudan University, China were tested.^1^ The East Asian sample consisted of 13 males and 16 females (*M*_age_ = 21.2, *SD* = 2.06, range: 19–26 years). All Chinese participants were native Mandarin speakers. The sample was part of a study in China (Zhou, Wang, & Zhou, 2014).^2^ We reanalyzed the data with new analyses to test our hypotheses, and we conducted cultural comparisons across data from the East Asian and European samples. These analyses have not been reported before.

#### Materials

Fifteen Chinese DRM lists were translated and adapted from Stadler, Roediger, and McDermott (1999). The BAS of each list was measured with a Chinese population in a pilot study based on the procedures in Roediger, Watson, McDermott, and Gallo (2001b). Participants were first given the Chinese words and were asked to write down the first word it brought to mind for each word. Then the connection strength between each study word (e.g., *garage, drive, road, Mercedes*) and its corresponding critical lure (e.g., *car*) was measured as the probability it elicited the critical lure. BAS was the average probability that the studied items elicited the critical lure. Hence, some DRM lists used in the East Asian sample were different from that of the European sample. Fifteen lists were pseudorandomly assigned to the self-reference (five lists; mean BAS = 0.19, *SD* = .04), other-reference (five lists; mean BAS = 0.20, *SD* = .04), and the neutral condition (five lists; mean BAS = 0.20, *SD* = .03), with BAS matched in the three conditions, *F*(2, 12) = 0.09, *p* = .91. The recognition list contained 15 critical lures (one per list), 45 studied items (three items per list), and 20 unrelated items. The studied items were from the first, sixth, and 10th position of each list.

#### Design and procedure

The experiment used a 3 (reference: self vs. other vs. neutral) × 2 (memory type: true vs. false) within-subjects design. The procedure of Experiment 2 was identical as in Experiment 1, except for the following differences. First, in the East Asian sample of Experiment 2, the name “Li Ming” (a frequently used name in Chinese textbooks) was used in the “other” condition. Second, in the neutral condition of Experiment 2, a red square appeared together with DRM words. Thus, the DRM words appeared together with either their own name or the name “Li Ming” or a red square in the study phase. Other steps in the procedure were exactly the same as in Experiment 1.

### Results and discussion

#### Recognition rates

The mean false recognition rate of unrelated items was 8.45% (95% CI [4%, 13%]), which was statistically lower than that of critical lures in any condition (*p*s < .001). We conducted a 3 (reference: self vs. other vs. neutral) × 2 (memory type: true vs. false) repeated-measures ANOVA on true and false recognition rates. The results were similar to those in Experiment 1. The main effect of reference was statistically significant, *F*(2, 56) = 25.45, *p* < .001, partial η^2^ = 0.48. Bonferroni comparisons showed that self-reference led to higher true and false recognition rates than both the other-reference (*p* < .001) and neutral conditions (*p* < .001). The main effect of memory type was not significant, *F*(2, 56) = 0.93, *p* = .34. No significant interaction between reference and memory type was found, either, *F*(2, 56) = 1.88, *p* = .16.

Results showed that the self-reference false memory effect was revealed in Experiment 2 as well. Specifically, the self-reference condition had significantly higher false memories than the other-reference condition did, *t*(28) = 3.19, *p* = .004, Cohen’s *d* = 0.61, and the neutral condition, *t*(28) = 2.45, *p* = .02, Cohen’s *d* = 0.46. The other-reference condition did not differ from the neutral condition in false recognition rates, *t*(28) = 0.96, *p* = .35. A typical SRE of true memories was also found. A paired-samples *t* test showed significant higher true memory rates in the self-reference condition than in the other-reference condition, *t*(28) = 7.23, *p* < .001, Cohen’s *d* = 1.46.

#### Net accuracy

Net accuracy was calculated for the self-reference, other-reference, and neutral conditions. Repeated-measures ANOVA showed that there was no significant effect of reference on net accuracy, *F*(2, 56) = 2.12, *p* = .13. Self-referencing (*M* = 0.51, 95% CI [0.49, 0.53]) resulted in similar memory net accuracy as in the other-referencing (*M* = 0.48, 95% CI [0.45, 0.50]) and neutral (*M* = 0.50, 95% CI [0.47, 0.53]) conditions. In an East Asian sample, it was also found that self-referencing did not improve the overall memory accuracy with the DRM paradigm.

Experiment 2 found comparable results in an East Asian sample as with a European sample in Experiment 1. Besides the difference in sample population, Experiment 2 used different DRM lists and a different other-referenced name. However we still found that self-referencing increased false memories than other-referencing did and also replicated the results that self-referencing did not impact net accuracy of memory.

#### Joint analyses on false memory

To explore whether the magnitude of the SR false memory effect differed statistically in European and East Asian samples, we combined data of the two samples and treated the sample as a between-subjects variable. A 2 (sample: European vs. East Asian) × 2 (reference: self vs. other) repeated-measures ANOVA was conducted on false recognition rates, where sample was between-subjects and reference was within-subjects. No statistical interaction between sample and reference was found, *F*(1, 66) = 0.07, *p* = .79, suggesting sample did not interact with the SR false memory effect. There was a main effect of reference, *F*(2, 66) = 23.50, *p* < .001, partial η^2^= 0.26. That is, in both the European and East Asian samples, self-referencing (*M* = 0.78, 95% CI [0.73, 0.83]) led to higher false memory rates than did other-referencing (*M* = 0.66, 95% CI [0.62, 0.71]; *p* < .001, Cohen’s *d* = 0.59). There was also a main effect of sample. East Asian participants (*M* = 0.79, 95% CI [0.73, 0.86]) had statistically more false memories than European participants did (*M* = 0.65, 95% CI [0.60, 0.71]), *F*(1, 66) = 11.57, *p* = .001, partial η^2^ = 0.15.

#### Joint analyses on net accuracy

A 2 (sample: European vs. East Asian) × 2 (reference: self vs. other) repeated-measures ANOVA on net accuracy was conducted. No statistical interaction effect between sample and reference was found, *F*(1, 66) = 0.75, *p* = .39. There was no main effect of sample, *F*(1, 66) = 0.59, *p* = .44, indicating no net accuracy differences in European (*M* = 0.51, 95% CI [0.48, 0.53]) and East Asian samples (*M* = 0.49, 95% CI [0.46, 0.52]). No main effect of reference was found, either, *F*(1, 66) = 0.99, *p* = .32, suggesting self-referencing (*M* = 0.51, 95% CI [0.49, 0.53]) did not impact net accuracy compared with other-referencing (*M* = 0.49, 95% CI [0.46, 0.52]) across our two samples. Although East Asian participants had different self-referenced true and false memory rates relative to the European sample, self-referencing had a very similar effect on net accuracy in the two samples.

Contrary to our prediction, there was no cultural difference in self-referenced false memories and net accuracies. To note, there are some differences in the experimental materials between the samples, so one needs to be cautious when interpreting the joint analyses. One difference is that the Chinese DRM lists were different from lists used in the European sample. The names used in the other-reference conditions of the two samples were also different, which was due to cultural difference, but maybe in future research the names can be more strictly comparable. Another difference is the number of items in the recognition test. Although the proportions of critical lure in the recognition test were comparable (20% vs. 19% in European and East Asian samples), the different number of studied items per list might impact the results. Nevertheless, the SR false memory effect is based on within-subject comparisons between self-reference and other-reference conditions. Those differences should not have impacted the main message of the current study.

## General discussion

The current study examined whether processing information in relation to oneself (i.e., self-referencing) increased false memories compared with processing information in relation to others (i.e., other-referencing). In two experiments with Eastern and Western samples, we found consistent results that self-referencing enhanced both false recognition rates of critical lures and true recognition rates of studied items in the DRM paradigm. Specifically, self-referencing impacted recollection but not familiarity of studied items and critical lures. In addition, we found that self-referencing did not increase the net accuracy compared with other-referencing and neutral conditions. The current findings support the theoretical view that false memories are not malfunctions of cognitive processes (e.g., Howe, 2011; Schacter, 2012), but that true and false memories may originate from similar memory mechanisms such as spreading activation and gist processing.

Indeed, the current findings can be readily explained by spreading activation theories of memory (Howe et al., 2009; Roediger et al., 2001a), in which relational processing or binding plays a key role. In this theoretical view, nodes (e.g., words, images) and associative links (i.e., relations or bindings) are the cognitive units of memory, and they make up the whole memory network (Anderson, 1983; Howe et al., 2009). When participants process the DRM words in relation to themselves, DRM items that share the same theme (i.e., critical lure) are more strongly linked to each other than when DRM words are processed in relation to others. Consequentially, memories for DRM words are better retained under self-referencing and theoretically, activation levels of DRM items in an associative memory network is increased by self-referencing since activation level determines memory rates (Anderson, 1983). Thus, more activation can be spread to related but nonpresented critical lures in a memory network, and hence memory rates for critical lures are higher in the self-reference condition. In short, self-referencing increases binding among concepts in a memory network, which leads to more activation spreading along the network to critical lures and this in turn increases false memory rates.

The SRE on both true and false memories supports fuzzy-trace theory as well. Previous research has shown that self-referencing improves relational processing (e.g., semantic relations) of studied items (Klein, 2012). According to FTT, semantic relations are represented as gist traces in memory (Brainerd & Reyna, 2005). Thus, self-referencing may boost gist processing of studied lists. Gist traces support not only correct recognition of studied items but also false recognition of nonpresented but meaning-consistent critical lures (Reyna, Corbin, Weldon, & Brainerd, 2016). As a result, self-referencing can increase both true recognition of studied items and false recognition of critical lures.

Based on the activation theories and FTT, we propose that cognitive processes/factors that increase mnemonic efficiency (by increasing relational binding among memory nodes or gist extraction of stimuli) may increase susceptibility to associative false memories. The following evidence supports the above proposition. First, our results here have already demonstrated that self-referencing as a superior mnemonic processing leads to high levels of false memories. Second, research has found that deeper processing of the information (e.g., defining an item), which is known to enhance true memories, produces more false memories than shallow processing does (e.g., counting the letters of a word; Rhodes & Anastasi, 2000; Thapar & McDermott, 2001). Third, survival processing (e.g., rating items’ relevance for a survival scenario) has been found to boost true memories (Nairne & Pandeirada, 2008; Nairne, Thompson, & Pandeirada, 2007); however, it has also been found to increase susceptibility to memory illusions (Howe & Derbish, 2010; Otgaar & Smeets, 2010). Fourth, individuals with highly superior autobiographical memories, who can accurately remember everyday details since midchildhood, are found to be more susceptible to suggestive false memories than are normally remembering adults (Patihis et al., 2013).

Other evidence has come from the development of relational binding and development of false memories. The ability of binding among stimuli develops with age. Research has found that people’s ability to bind isolated parts of pictures improves from childhood to adulthood (Sluzenski, Newcombe, & Kovacs, 2006). Lee, Wendelken, Bunge, and Ghetti (2016) have found that item–item and item–time bindings improve gradually from 8 years of age to adulthood. Interestingly, there is a similar developmental pattern (i.e., the development reversal) of false memory formation from children to adults. That is, children exhibit fewer false memories generated from associative lists such as DRM lists than adults do (e.g., Brainerd et al., 2008a; Howe et al., 2009; Otgaar, Howe, Brackmann, & Smeets, 2016). More intriguingly, older adults exhibit the phenomenon of hyperbinding, in which they make too many associations (Campbell, Hasher, & Thomas, 2010), creating more associative false memories than young adults do (Devitt & Schacter, 2016; Schacter, Koutstaal, & Norman, 1997). Similarly, as predicted by FTT, gist sensitivity increases with age, and thus false memory increases with age (Brainerd et al., 2008a). Taken together, from a developmental perspective, binding ability and gist extraction ability increase with age from childhood until later adulthood, and susceptibility to associative false memories increases correspondingly.

Relational binding is obviously significant for human memory since it allows individuals to bind features of an experience into an integrated episodic memory (Eichenbaum & Cohen, 2004), and so are self-referencing, deep processing, and survival processing functional for human memory. However, those cognitive functions inevitably lead to unwanted false memories under certain circumstances. Thus, false memories reflect the adaptive nature of memory in the sense that they originate from efficient functioning of the memory system instead of being malfunctions of the memory system (see also Howe, 2011; Howe & Derbish, 2010; Schacter, 2012).

Contrary to our prediction, we did not find any cultural difference in the SR false memory effect and net accuracy. This suggests that the SRE on true and false memories is not sensitive to cultures and may represent a universal memory mechanism. One possible explanation is that spreading activation in a memory network is automatic (Howe, 2005, 2006; Howe et al., 2009; Roediger et al., 2001a) and thus not impacted by cultural backgrounds. The automaticity of memory activations implies that activations of critical lures cannot be controlled by consciousness, such as consciously making references to others. Another reason why we did not find a cultural difference may be due to the identity of others used in the other-reference condition. Both “Trump” and “Li Ming” were familiar, but not intimate others for our participants. Zhu et al. (2007) have found that when East Asians make references to their mothers, a similar neural pattern as SRE was found. If we use participants’ mothers in the other-reference condition in European and East Asian samples, we may find a cultural difference on SR false memories. Further research is needed regarding cultural difference on SRE.

In conclusion, we found a self-reference false memory effect that processing information in relation to oneself led to more false memories than processing information in relation to others (e.g., Trump). The SR false memory effect was stable across Western and Eastern population samples, with identical effect sizes, Cohen’s *d* around 0.6. What’s more, self-referencing did not increase memory net accuracy across two samples. Based on spreading activation theories of memory, we propose that cognitive processes/factors that increase mnemonic efficiency may increase susceptibility to associative false memories, which reflects the adaptive nature of memory.

## Electronic supplementary material

ESM 1 (DOCX 25 kb)

## Footnotes

1 Post hoc power analysis indicated that the power of Experiment 2 was 0.87 with sample size *n* = 29.

2 We achieved part of the data in this work, which is written in Chinese, to conduct some new analyses.
