# Supplementary material for: A self-reference false memory effect in the DRM paradigm: Evidence from Eastern and Western samples
Source: Mem Cognit. 2018 Aug 23;47(1):76–86. doi: 10.3758/s13421-018-0851-3 (PMC6351515; doi:10.3758/s13421-018-0851-3)
Supplement: Supplementary file 1 — (DOCX 25 kb) [file 13421_2018_851_MOESM1_ESM.docx]

Table 1

*Mean true and false recognition rates in different reference conditions with 95% confidence intervals (Experiment 1; n = 39)*

|  |  | Reference conditions | | |
| --- | --- | --- | --- | --- |
|  |  | Self-reference | Other-reference | Neutral |
| Memory type | True memory | 0.70 [0.65, 0.75] | 0.62 [0.55, 0.70] | 0.65 [0.58, 0.71] |
|  | False memory | 0.71 [0.64, 0.78] | 0.60 [0.54, 0.66] | 0.63 [0.55, 0.71] |

Table 2

*Mean true and false recognition rates in different reference conditions with 95% confidence intervals (Experiment 2; n = 29)*

|  |  | Reference conditions | | |
| --- | --- | --- | --- | --- |
|  |  | Self-reference | Other-reference | Neutral |
| Memory type | True memory | 0.87 [0.84, 0.91] | 0.66 [0.60, 0.73] | 0.75 [0.69, 0.80] |
|  | False memory | 0.86 [0.80, 0.91] | 0.73 [0.66, 0.81] | 0.77 [0.69, 0.84] |
